# Activation of CD40 with Platelet Derived CD154 Promotes Reactive Oxygen Species Dependent Death of Human Hepatocytes during Hypoxia and Reoxygenation

**DOI:** 10.1371/journal.pone.0030867

**Published:** 2012-01-25

**Authors:** Ricky H. Bhogal, Christopher J. Weston, Stuart M. Curbishley, David H. Adams, Simon C. Afford

**Affiliations:** Centre for Liver Research, School of Infection and Immunity, Institute of Biomedical Research, The Medical School, The University of Birmingham, Edgbaston, Birmingham, United Kingdom; Institut National de la Santé et de la Recherche Médicale - Institut Cochin, France

## Abstract

**Background:**

Hypoxia and hypoxia-reoxygenation (H-R) are pathogenic factors in many liver diseases that lead to hepatocyte death as a result of reactive oxygen species (ROS) accumulation. The tumor necrosis factor super-family member CD154 can also induce hepatocyte apoptosis via activation of its receptor CD40 and induction of autocrine/paracrine Fas Ligand/CD178 but the relationship between CD40 activation, ROS generation and apoptosis is poorly understood. We hypothesised that CD40 activation and ROS accumulation act synergistically to drive human hepatocyte apoptosis.

**Methods:**

Human hepatocytes were isolated from liver tissue and exposed to an *in vitro* model of hypoxia and H-R in the presence or absence of CD154 and/or various inhibitors. Hepatocyte ROS production, apoptosis and necrosis were determined by labelling cells with 2′,7′-dichlorofluorescin, Annexin-V and 7-AAD respectively in a three-colour reporter flow cytometry assay.

**Results:**

Exposure of human hepatocytes to recombinant CD154 or platelet-derived soluble CD154 augments ROS accumulation during H-R resulting in NADPH oxidase-dependent apoptosis and necrosis. The inhibition of c-Jun *N*-terminal Kinase and p38 attenuated CD154-mediated apoptosis but not necrosis.

**Conclusions:**

CD154-mediated apoptosis of hepatocytes involves ROS generation that is amplified during hypoxia-reoxygenation. This finding provides a molecular mechanism to explain the role of platelets in hepatocyte death during ischemia-reperfusion injury.

## Introduction

Hepatocyte death is central to most liver diseases and is frequently associated with tissue hypoxia. Hypoxia can also occur as a result of ischaemia associated with hepatic surgery or liver transplantation where reoxygenation follows hypoxia and may further exacerbate tissue injury and promote liver inflammation. Central mediators of hepatocyte cell death during hypoxia are the reactive oxygen species (ROS) [1]. ROS, which include superoxide anions, hydrogen peroxide, hypohalus acid and hydroxyl radicals, accumulate in the many liver diseases and have also been implicated in the preservation/reperfusion injury that follows liver transplantation [2].

Under physiological conditions hepatocytes exist in a relatively hypoxic micro-environment [3] which promotes the generation of ROS. Consequently hepatocytes have several cellular mechanisms, including high intracellular glutathione levels, which counteract increases in ROS and thereby limit parenchymal injury [4], [5]. It has long been assumed that extracellular ROS, released by Kupffer cells (KCs) and infiltrating neutrophils are the main contributors to hepatocyte injury [6]. However, we recently reported that intracellular ROS can also regulate human hepatocyte cell death during hypoxia and hypoxia-reoxygenation (H-R) [2]. In this micro-environment, hepatocytes will be exposed to an array of pro-inflammatory mediators including cytokines which may augment intracellular ROS production and amplify liver injury.

CD40 and its ligand CD154, members of the tumor necrosis factor-(TNF) receptor/ligand superfamily are known to regulate hepatocyte apoptosis [7] and have been implicated in hepatocyte death during hypoxia [8]. CD40 is a type I trans-membrane protein receptor and expressed as a trimeric complex upon the cell surface of many cells including hepatocytes, endothelial cells and cholangiocytes. CD154, a type II trans-membrane protein, is expressed by CD4^+^ T-lymphocytes, macrophages, mast cells and basophils. It is also expressed by platelets and released in a soluble form following platelet activation. Studies in CD40 and CD154 deficient mice have implicated this pathway in hypoxic liver injury [8], [9]. The mechanism was assumed to involve defective CD40∶CD154 priming of T-lymphocytes. However, we have previously reported that activation of CD40 on hepatocytes or cholangiocytes can induce Fas Ligand (FasL/CD178) expression and autocrine/paracrine Fas-mediated apoptosis [7], [10]. CD40∶CD154 interactions have also been shown to mediate ROS accumulation in the WEHI231 lymphoid cell line [11] leading us to hypothesise that CD40 activation could be implicated in ROS-mediated hepatocyte death.

Following ligation by trimeric CD154, CD40 is activated and internalized. CD40 has no active intracellular kinase domain and binds to members of the tumor necrosis factor receptor-associated factor (TRAF) family which are responsible for activating downstream signaling pathways. In lymphoid cells, TRAFs recruit the cystolic enzyme nicotinamide adenine dinucleotide phosphate oxidase (NADPH oxidase) to the plasma membrane providing a potential mechanism to generate ROS [11]. We now report that in primary human hepatocytes, CD40 ligation leads to NADPH-dependent intracellular ROS generation and hepatocyte death in hypoxic conditions demonstrating for the first time how TNF receptor activation and ROS can co-operate to mediate epithelial cell injury in chronic inflammation and hypoxia/reperfusion injury.

## Materials and Methods

### Ethics Statement

Liver tissue was obtained from surgical procedures carried out at the Queen Elizabeth Hospital, Birmingham, UK. Ethical approval for the study was grant by the Local Research Ethics Committee (LREC) (Leicestershire and Rutland Ethics Committee - reference number 06/Q702/61). Informed written consent was obtained from all participants involved in the study.

### Human Hepatocyte Isolation

Liver tissue was obtained from fully consenting patients undergoing transplantation, hepatic resection for liver metastasis, hepatic resection for benign liver disease or normal donor tissue surplus to surgical requirements. Human hepatocytes were isolated using a method that we have described previously [2], [12].

### Model of hypoxia and H-R injury

We used a model of *in vitro* hypoxia and H-R that we have described previously [2]. In experiments involving the NADPH oxidase inhibitor, DPI; JNK inhibitor SP600125, or p38 inhibitor, PD169316, all reagents were made fresh as stock solutions and added using the correct dilutional factor to the relevant experimental wells. Specifically, 10 µg DPI (Sigma) was dissolved in molecular grade dimethyl sulfoxide (DMSO), 10 µg rotenone (Sigma) was dissolved in chloroform (Sigma), 50 µg SP600125 (Sigma) was dissolved in DMSO and 50 µg PD169316 (Sigma) was dissolved in DMSO and were diluted appropriately to give working concentrations of 10 µM, 2 µM, 10 µM and 10 µM respectively. In experiments using inhibitors/antioxidants, solvent alone wells were used to control for vehicle effects. In experiments using inhibitors/antioxidants hepatocytes were pre-treated with agents for up to 4 hours before placement of the cells into normoxia and hypoxia. For H-R experiments fresh inhibitor/antioxidants were added at the time of placement into reoxygenation. Recombinant human soluble CD154 (1 µg/mL, Enzo Life Sciences, UK) and 1 µg/mL Cross-linker for Ligands (Enzo Life Sciences, UK) were added to cells at the time of entry into hypoxia or H-R. Where cells had been pre-treated with inhibitors/antioxidants CD154 and Cross-linker for Ligands were added after 4 hours.

### Determination of Human Hepatocyte CD40 Expression and FasL Expression

Following appropriate incubation of human hepatocytes within normoxia, hypoxia and H-R, cells were trypinised and washed in FACs buffer (Phosphate-buffered saline (PBS) pH 7.2 with 10% v/v heat inactivated foetal calf serum (Gibco). For CD40 expression, cells were then incubated with anti-human CD40 antibody that was conjugated to the APC fluorophore (1∶100 dilution; Caltag, UK) for 45 min at 4°C. Mouse IgG1-APC (1∶100 dilution; Caltag, UK) was used as a negative control. For FasL expression, cells were then incubated with anti-human FasL antibody that was conjugated to the FITC fluorophore (1∶100 dilution; Abcam, UK) for 45 min at 4°C. Mouse IgG2a-FITC (1∶100 dilution; Abcam, UK) was used as a negative control. Following this cells were washed in FACs buffer and resuspended in PBS, pH 7.2. At least 20,000 events were recorded within the gated region of the flow cytometer for each human hepatocyte cell preparation in each experimental condition. Only the cells within the gated region were used to calculate Mean Fluorescence Intensity (MFI) as described in our previous study [2].

### Determination of Human Hepatocyte ROS Accumulation, Apoptosis and Necrosis

ROS production, apoptosis and necrosis were determined using a three-colour reporter assay system as previously described [2]. At least 20,000 events were recorded within the gated region of the flow cytometer for each human hepatocyte cell preparation in each experimental condition. Only the cells within the gated region were used to calculate MFI.

### Platelet isolation and activation

Platelets were isolated by modifying a previously published method from fully consenting healthy individuals [13]. Platelet-rich plasma (PRP) was prepared by centrifuging 5 mL of heparinised blood for 5 min at 300× *g*. Following this the PRP was withdrawn and the platelet count adjusted to 1×10^6^ platelets/mL in Williams E media. Platelets were then seeded onto plastic. Following 30 min incubation at room temperature the platelets had settled, become activated and spread to form a confluent monolayer. The Williams media was then aspirated and passed through a 0.2 µm syringe filter. The PCM was aliquoted and frozen at −70°C until required. Media was thawed and added to cells as required.

### Determination of Soluble CD154 and Immunomagnetic Depletion of CD154 from PCM

To confirm the presence and quantity of CD154 in the PCM levels were quantified by ELISA and platelet-derived CD154 was removed via immuno-magnetic depletion. Briefly, soluble human CD154 was measured in both PCM and CD154-depleted PCM using a sandwich ELISA kit (Peprotech, UK). For immunomagnetic depletion of CD154, anti-human CD154 antibody (1 µg/mL; Abcam, UK) was complexed to pan-mouse IgG magnetic Dynabeads (Invitrogen, Paisley, UK) for 90 min at room temperature. Following this, the antibody-magnetic bead complexes were extensively washed. Complexes were then incubated with PCM for a further 90 min at room temperature. Finally, magnetic beads were retrieved from solutions and this media was subsequently used in experiments as CD154-depleted PCM.

### Statistical Analysis

All data are expressed as mean ± S.E. Statistical comparisons between groups were analysed by Student's *t* test. All differences were considered statistically significant at a value of p<0.05.

## Results

### CD40 expression in human hepatocytes during hypoxia and H-R

**Figure 1:**
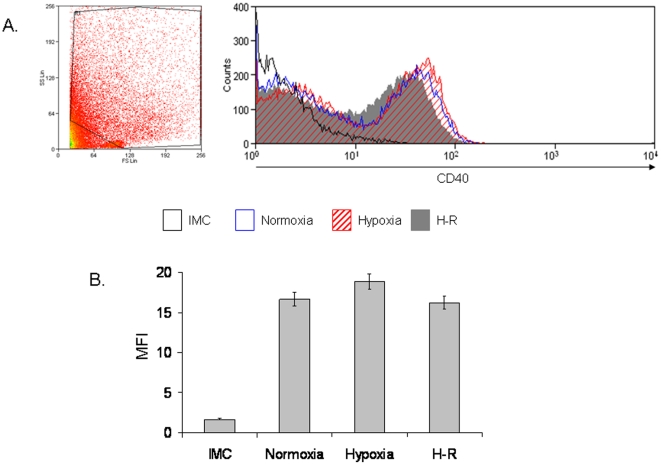
CD40 Expression on Primary Human Hepatocytes. Figure 1a demonstrates a representative flow cytometry plot of CD40 expression on primary human hepatocytes during normoxia, hypoxia and H-R. The plot on the left hand side represents a typical forward scatter (FS) versus side scatter (SS) plot of primary human hepatocytes. The FS versus SS plots shown is from the H-R sample of a liver preparation but similar plots were obtained during normoxia and hypoxia (data not shown). Figure 1b shows a bar chart with the pooled data of three separate experiments illustrating the level of CD40 expression on primary human hepatocytes. Data are expressed as MFI and readings are based upon values taken from cells within the gated region in Figure 1a.

Primary human hepatocytes constitutively expressed CD40 on the cell membrane as previously reported [14] but this did not increase in response to hypoxia or H-R *in vitro* (Figure 1).

### CD40∶CD154 stimulates ROS accumulation in human hepatocytes in an NADPH-dependent manner and regulates apoptosis and necrosis

**Figure 2:**
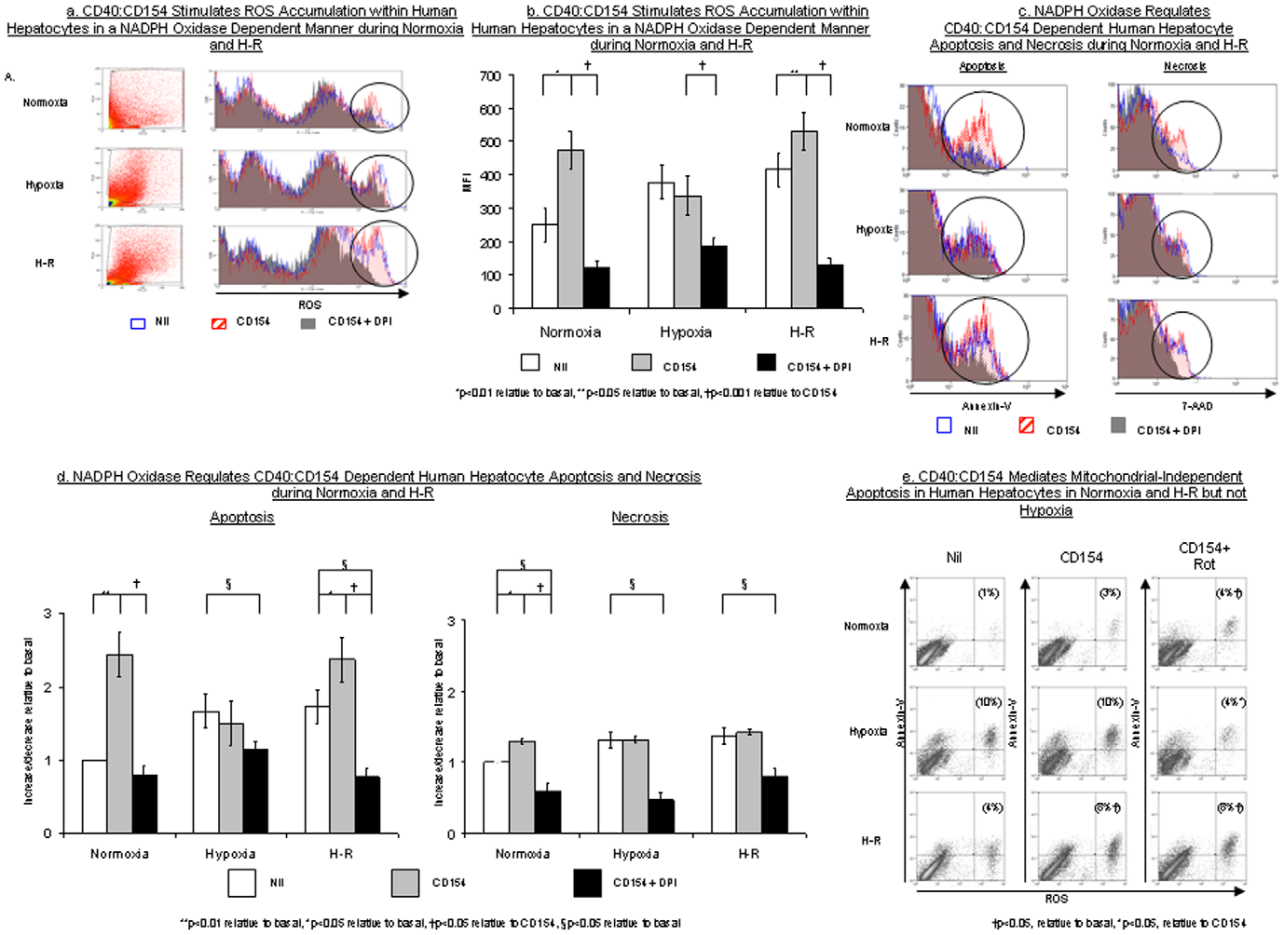
CD40∶CD154 regulates ROS accumulation, apoptosis and necrosis within human hepatocytes in a NADPH Oxidase dependent manner during normoxia and H-R. Figure 2a demonstrates representative flow cytometry plots to illustrate the effect of CD154 (hatched red) and CD154 in presence of DPI (solid grey) upon human hepatocyte ROS accumulation during normoxia, hypoxia and H-R. Typical FS versus SS plots of primary human hepatocytes during normoxia, hypoxia and H-R are shown to the left of each flow cytometric plot. The FS versus SS plots shown is from the H-R alone sample of a liver preparation but similar plots were obtained during normoxia and hypoxia (data not shown). The areas of interest on the flow cytometric plots are marked by the vertical ellipses. The area on the left of each ellipse represents cell debris. Cell debris is included within the plot as human hepatocytes vary considerably in size and therefore to include all viable human hepatocytes in the analysis a large gate is required on the flow cytometer, this by necessity includes the cell debris. Figure 2b. shows a bar chart with the pooled data of three separate experiments illustrating the effects of CD154 and CD154+DPI upon human hepatocytes ROS accumulation. Data is expressed as MFI and readings are based upon values taken from cells within the gated region shown in Figure 2a. Data are expressed as mean ± S.E. (*p<0.01 relative to basal, **p<0.05 relative to basal, †p<0.001 relative to CD154). Figure 2c demonstrates representative flow cytometry plots to illustrate the effect of CD154 (hatched red) and CD154 in presence of DPI (solid grey) upon human hepatocyte apoptosis and necrosis during normoxia, hypoxia and H-R. Again, the area of interest within the flow cytometric plots is marked by the vertical ellipse. The same gate has been applied to primary human hepatocytes for these plots as those shown in Figure 2a. Figure 2d. shows a bar chart with the pooled data of three separate experiments illustrating the effects of CD154 and CD154+DPI upon human hepatocytes apoptosis and necrosis during normoxia, hypoxia and H-R. Data is expressed as increase/decrease relative to basal, where basal refers to the level of apoptosis or necrosis during normoxia alone. Data are expressed as mean ± S.E. (**p<0.01 relative to basal, *p<0.05 relative to basal, †p<0.05 relative to CD154, §p<0.05 relative to basal). Figure 2e. Human hepatocytes were treated with CD154 or CD154 following pre-treatment with rotenone (Rot) during normoxia, hypoxia and H-R. The percentage of cells staining with both the ROS probe DCF and apoptotic marker, Annexin-V, were assessed by flow cytometry. The percentage of human hepatocytes that stain for both DCF and Annexin-V are shown in parentheses. Data are representative of 3 separate experiments (†p<0.05 relative to basal, *p<0.05 relative to CD154).

We have recently reported that intracellular ROS accumulate in hypoxic human hepatocytes and that this is increased further during H-R [2]. Here we investigated whether CD154 could augment human hepatocyte ROS production during normoxia, hypoxia and H-R. Trimeric recombinant human CD154 significantly increased intracellular ROS accumulation within human hepatocytes during normoxia and H-R but not hypoxia. Because CD40∶CD154 has been shown to mediate ROS accumulation via TRAF-dependent recruitment of the flavoenzyme NADPH oxidase in lymphoid cell lines [11] we used the specific NADPH oxidase inhibitor Diphenyliodonium (DPI) to assess the involvement of this pathway in ROS generation by human hepatocytes. DPI reduced hepatocyte CD154 mediated ROS production during normoxia, hypoxia and H-R (Figure 2a & 2b).

The increased levels of intracellular ROS induced by CD154 stimulation, during normoxia and H-R, increased human hepatocyte apoptosis and to a lesser extent necrosis (Figure 2c & 2d) and this were reduced by pre-treatment with DPI. CD154 also significantly increased human hepatocyte necrosis during normoxia and H-R that was inhibited by DPI demonstrating that that the CD40∶CD154∶ROS signaling pathway is NADPH-dependent and results in hepatocyte death.

Mitochondrial ROS has been implicated in hepatocyte apoptosis during hypoxia and H-R [2] leading us to test the effect of inhibiting mitochondrial ROS production upon CD154 mediated apoptosis with the complex I inhibitor, rotenone. Inhibiting mitochondrial ROS production during normoxia and H-R did not reduce human hepatocyte apoptosis in response to CD154 treatment but did reduce apoptosis during hypoxia (Figure 2e).

### JNK and p38 regulate apoptotic but not necrotic cell death in human hepatocytes

We next investigated whether CD40∶CD154 generated ROS and hepatocyte death was associated with activation of mitogen-activated protein kinases (MAPKs). MAPKs consist of three sequentially activated kinase modules, namely mitogen-activated protein kinase kinase kinases (MAPKKK/MAP3K) followed by the mitogen-activated protein kinase kinases (MAPKK/MEKK) and finally the MAPKs. The MAPKs superfamily consists of three separate sub-families; extra-cellular regulated protein kinase (ERK), c-Jun *N*-terminal kinase (JNK) and p38. ERK is generally involved in the regulation of cell proliferation and differentiation, whereas JNK and p38 are involved in the regulation of cell death [15].

**Figure 3:**
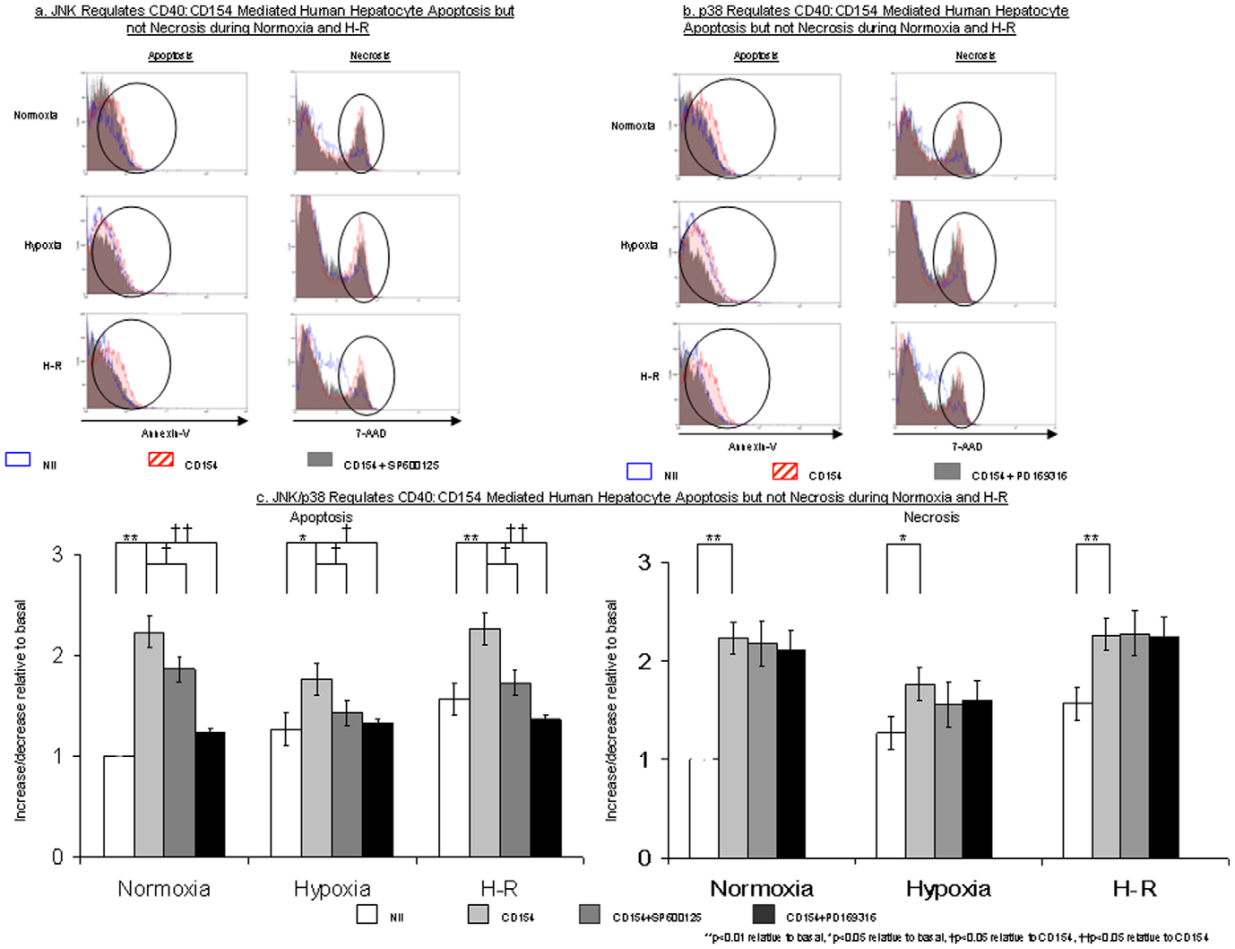
JNK and p38 regulate CD40∶CD154 mediated human hepatocyte apoptosis but not necrosis. Figure 3a show representative flow cytometry plots to illustrate the effect of the JNK inhibitor SP600125 upon CD40∶CD154 mediated apoptosis and necrosis during normoxia, hypoxia and H-R. The gate used to analyse primary human hepatocytes is the same as that shown in Figures 1 & 2. The area of interest within the flow cytometric plots are marked by the vertical ellipses. Figure 3b illustrates the effects of the p38 inhibitor P169316 upon CD40∶CD154 mediated apoptosis and necrosis during normoxia, hypoxia and H-R. Figure 3c shows a bar chart with the pooled data of three separate experiments illustrating the effects of both SP600125 and PD169316 upon CD40∶CD154 mediated human hepatocyte apoptosis and necrosis during normoxia, hypoxia and H-R. Data are expressed as increase/decrease relative to basal, where basal refers to the level of apoptosis or necrosis during normoxia alone. Data are expressed as mean ± S.E. (**p<0.01 relative to basal, *p<0.05 relative to basal, †p<0.05 relative to CD154, ††p<0.05 relative to basal).

Unfortunately, primary human hepatocytes are not amenable to siRNA transfection or protein labeling [16], [17], [18]. Therefore to investigate the effects of JNK and p38 inhibition upon CD154∶ROS mediated human hepatocyte apoptosis we used specific JNK and p38 inhibitors [19], [20], [21]. Inhibition of JNK and p38 in hepatocytes using the specific inhibitors SP600125 and PD169316 had no effect on ROS accumulation during normoxia, hypoxia and H-R (data not shown) whereas they significantly decreased CD40∶CD154 mediated human hepatocyte apoptosis during normoxia, hypoxia and H-R (Figure 3a, 3b & 3c). Inhibition of p38 had a greater effect on hepatocyte apoptosis than JNK inhibition. Neither inhibitor reduced CD154 mediated hepatocyte necrosis under any of the experimental conditions (Figure 3). This suggests that CD40∶CD154 mediated human hepatocyte apoptosis during normoxia and H-R is ROS, JNK and p38-dependent whilst necrosis is ROS-dependent but MAPK-independent.

### CD40∶CD154∶NADPH oxidase interactions stimulate hepatocyte Fas ligand expression

**Figure 4:**
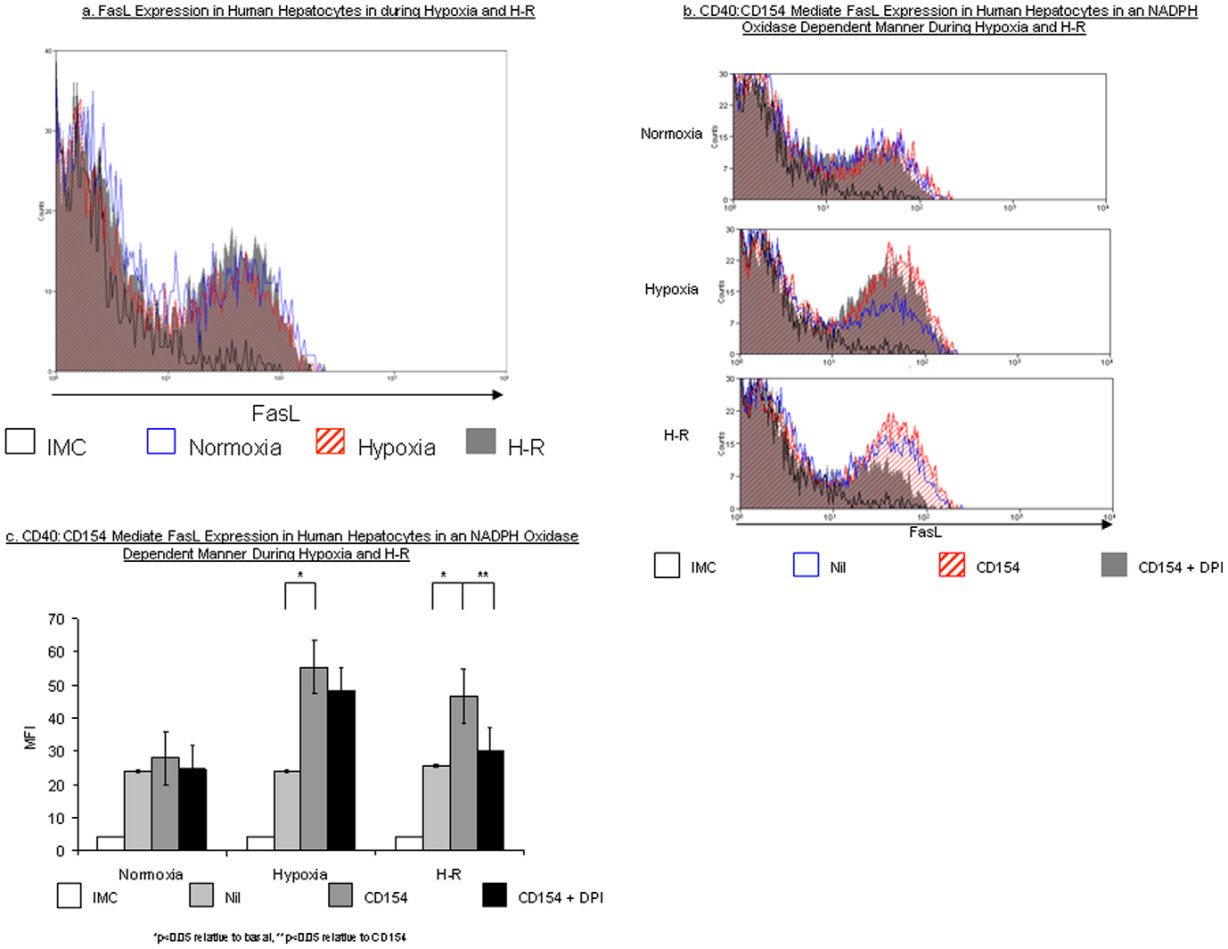
FasL Expression on Primary Human Hepatocytes. Figure 4a demonstrates a representative flow cytometry plot of FasL expression on primary human hepatocytes during normoxia, hypoxia and H-R. The gate used to analyse primary human hepatocytes is the same as that shown in Figures 1 & 2. Figure 4b. demonstrates representative flow cytometry plots to illustrate the effect of CD154 (hatched red) and CD154 in presence of DPI (solid grey) upon human hepatocyte FasL expression during normoxia, hypoxia and H-R. Figure 4c. shows a bar chart with the pooled data of three separate experiments illustrating the effects of CD154 upon human hepatocyte FasL expression during normoxia, hypoxia and H-R. Data are expressed as MFI and readings are based upon values taken from cells within the gated region in Figure 1a. Data are expressed as mean ± S.E. (*p<0.05 relative to basal, **p<0.05 relative to CD154).

Fas is a cell surface receptor of the TNF Receptor superfamily which, when activated by FasL, induces apoptosis on a wide range of cells including hepatocytes [7]. Because we have previously reported the CD40 activation on human hepatocytes results in Fas-mediated apoptosis, we investigated whether hypoxia or H-R alone or in association with CD154 induced FasL expression. Neither hypoxia nor H-R increased hepatocyte cell surface FasL expression (Figure 4a). However, CD40∶CD154 increased FasL expression during hypoxia and H-R, with a greater effect during hypoxia. Inhibiting NADPH oxidase function with DPI reduced CD40∶CD154 mediated FasL expression during hypoxia and H-R, with a greater effect noted during H-R (Figure 4b & 4c).

### Platelet-derived soluble CD154 mediates hepatocyte apoptosis and necrosis

Finally, because platelets are the source for 95% of circulating soluble CD154 and activated platelets may be found in the liver parenchyma during damage [22] we determined whether activated platelets may be a potential source of functionally active CD154 during hypoxia and H-R.

**Figure 5:**
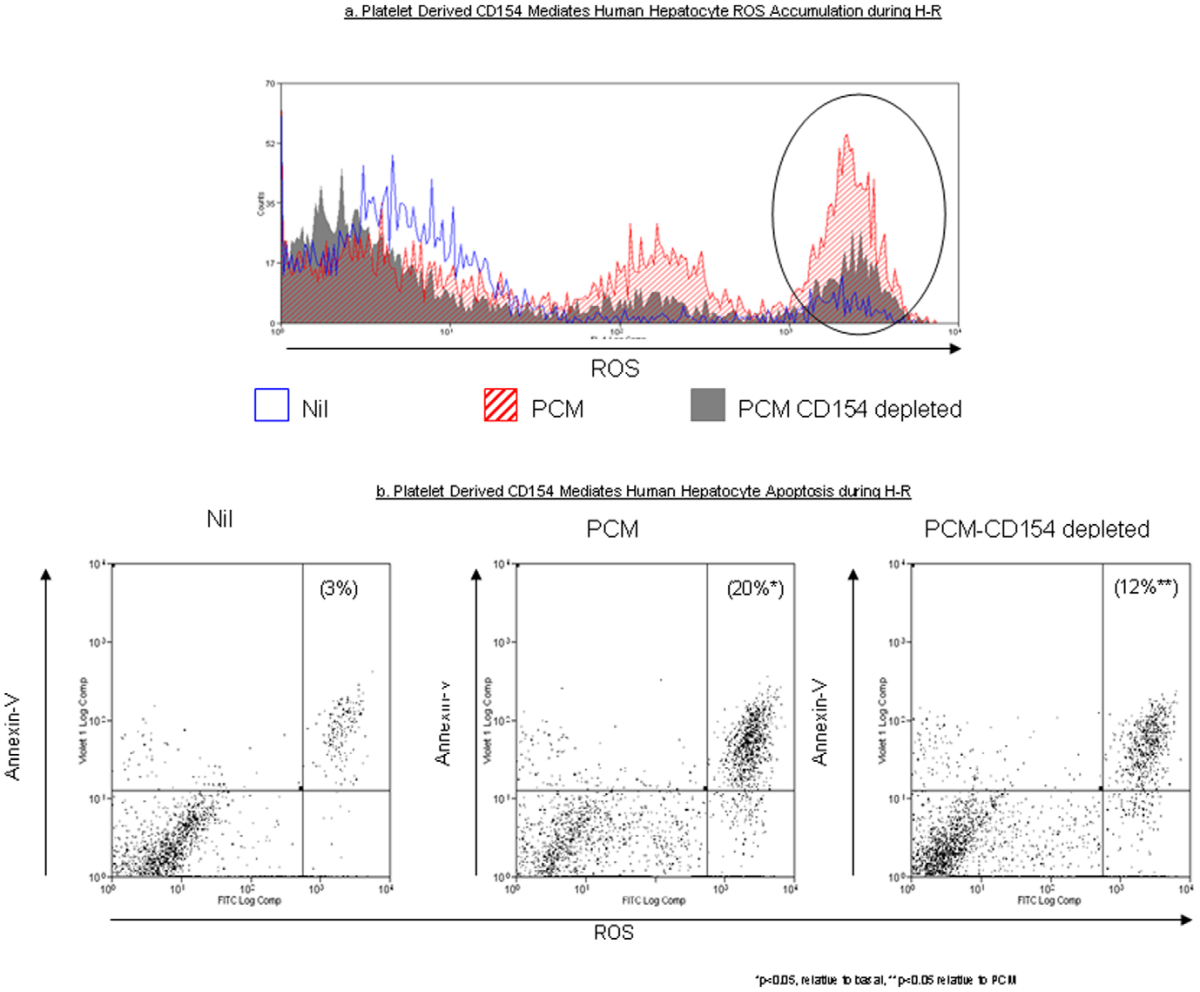
The effects of PCM and PCM CD154-depleted upon human hepatocyte ROS accumulation and cell death. Figures 5a demonstrates representative flow cytometry plots to illustrate the effect of PCM (hatched red) and PCM CD154-depleted (solid grey) upon human hepatocyte ROS accumulation during H-R. The gate used to analyse primary human hepatocytes is the same as that shown in Figures 1 & 2. The area of interest within the flow cytometry plots is marked by the vertical ellipse. The area on the left of each ellipse again represents cell debris. Figure 5b, human hepatocytes were treated with PCM or PCM CD154-depleted during H-R and the percentage of cells staining with both the ROS probe DCF and apoptotic marker, Annexin-V, were assessed by flow cytometry.. The percentage of human hepatocytes that stain for both DCF and Annexin-V are shown in parentheses. Data is representative 3 separate experiments (*p<0.05 relative to basal, **p<0.05 relative to PCM).

Immuno-magnetic depletion reduced soluble CD154 released by activated platelets by >70% (Platelet Conditioned Media (PCM) soluble CD154 concentration 2251±310 pg/mL; CD154-depleted PCM CD154 concentration 590±79 pg/mL). PCM increased hepatocyte intracellular ROS accumulation during H-R (Figure 5a). When CD154 was depleted from PCM, intracellular ROS accumulation was significantly reduced during H-R. PCM increased human hepatocyte apoptosis during H-R mirroring the increasing in intracellular ROS (Figure 5b). However, in line with the decrease in ROS seen during H-R, CD154-depleted PCM decreased human hepatocyte apoptosis during H-R (Figure 5b).

## Discussion

Tissue hypoxia is a feature of hepatic inflammation and disease [23] and we have recently shown the accumulation of intracellular ROS is a critical mediator of hepatocyte death during hypoxia and H-R [2]. In the present report we show that ROS accumulation in human hepatocytes during hypoxia and H-R is increased by CD154 mediated activation of hepatocyte CD40 resulting in increased cell death. This link between CD40 activation, intracellular ROS generation and cell death has implications for other inflammatory diseases characterised by local hypoxia.

CD40∶CD154 receptor-ligand interactions have been associated with parenchymal injury during hypoxia and H-R in many organs [8], [24], [25] and disruption of CD40∶CD154 signaling in mice improves liver function following hypoxia [8]. However until now it was thought that CD40∶CD154 mediates its actions through effects on T-lymphocyte priming whereas our data suggest another mode of action through direct effects on hepatocyte survival. We have previously reported that CD40 activation mediates Fas dependent apoptosis in human hepatocytes through induction of autocrine FasL expression [7]. We now demonstrate that CD40∶CD154 can also mediate human hepatocyte apoptosis and necrosis in a NADPH Oxidase∶ROS-dependent manner. This introduces a previously unknown CD40 dependent pathway to cell death and implicates CD40 in the regulation of cell survival during tissue hypoxia and H-R.

Our data also support an important role for intrinsic ROS accumulation in hepatocytes as opposed to the current model which suggests that ROS is derived from leukocytes [6]. CD40 activation did not increase ROS accumulation or cell death in human hepatocytes during hypoxia and this is probably a consequence of the increased antioxidant levels induced in hepatocytes during hypoxia [26]. This may also explain why FasL has little effect during hypoxia (see below). Furthermore, hypoxia alone did not increase hepatocyte cell surface CD40 implying that the lack of effect of CD154 during hypoxia was probably a consequence of the modulation of intracellular signaling pathways by antioxidants rather than changes in CD40 receptor bioavailability.

**Figure 6:**
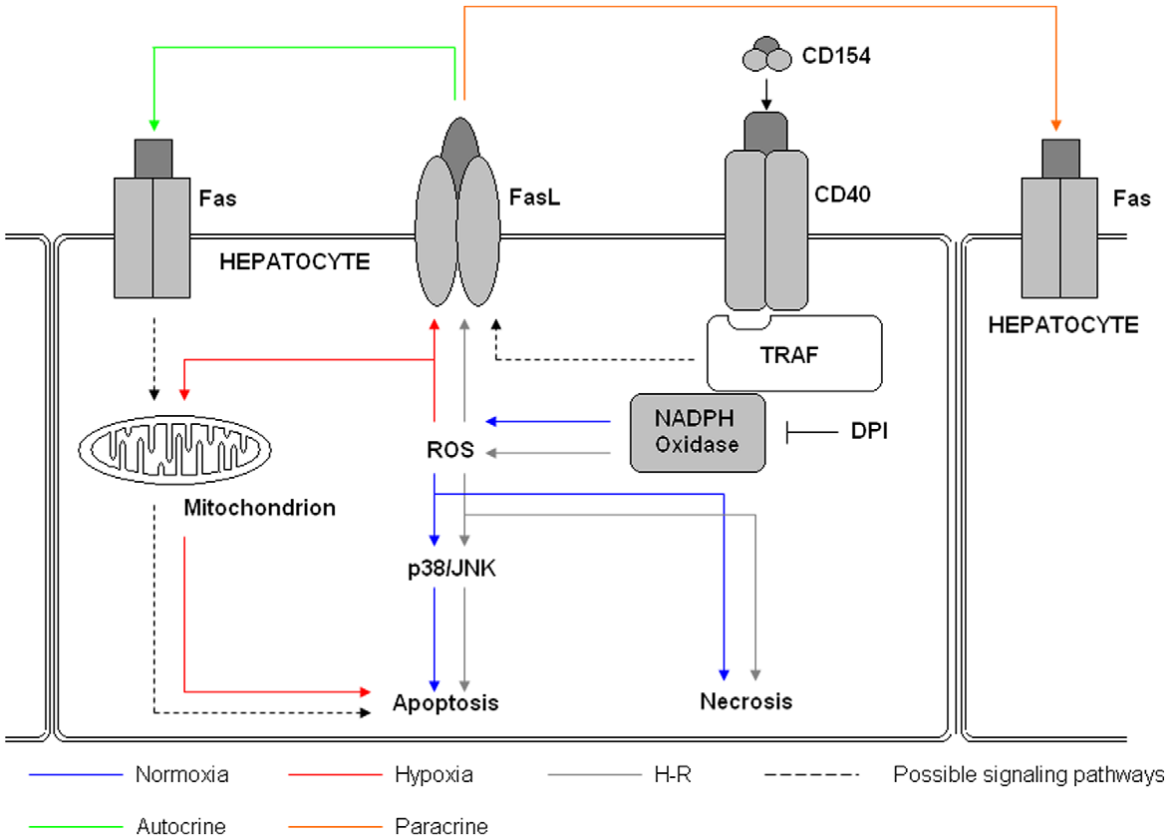
Proposed mechanism of CD40-CD154 mediated apoptosis and necrosis in human hepatocytes during normoxia, hypoxia and H-R. During normoxia (blue arrows) activation of CD40 expressed upon human hepatocyte by CD154 results in the translocation of the TRAF adaptor molecules to the cell membrane. The TRAF molecules are then responsible for the recruitment of the flavoenzyme NADPH Oxidase to the CD40∶TRAF complex. Here NADPH Oxidase can induce the production of ROS, primarily in the form of hydrogen peroxide. This process can be inhibited by the NADPH Oxidase inhibitor DPI. The resultant accumulation of ROS can result directly in necrotic cell death or it can activate the MAPK members, JNK and p38, to induce human hepatocyte apoptosis. However, during hypoxia (red arrows) CD40 activation on human hepatocytes does not result in ROS accumulation but can induce FasL expression via mechanisms that NADPH Oxidase-dependent and ROS-independent (dotted black line). This increased FasL expression can result in autocrine (green arrow) and/or paracrine (orange arrows) Fas-mediated apoptosis. Due to the increases in intracellular antioxidants levels during hypoxia CD154 does not increase apoptosis. However, mitochondrial ROS does contribute to apoptosis during hypoxia. During H-R, CD40 activation can lead to NADPH Oxidase-ROS-JNK/p38 dependent apoptosis, NADPH Oxidase-ROS-dependent necrosis and NADPH Oxidase∶ROS∶FasL expression with resultant Fas-mediated apoptosis. Figure 6 highlights the importance of local microenvironment in shaping the effects of CD40∶CD154.

Our data clearly show that increases in intracellular ROS induced by CD154 are dependent upon NADPH Oxidase, this is consistent with previous studies implicating NADPH in ROS accumulation in hypoxic human hepatocytes during normoxia, hypoxia and H-R [2]. Studies in WEHI 231 cells and endothelial cells have shown that following activation, CD40 recruits NADPH Oxidase to the plasma membrane via a mechanism involving TRAF3 [11], [27], [28], [29]. Once at the plasma membrane the p40^phox^ subunit of NADPH oxidase can generate ROS leading to activation of a number of downstream cell death signaling pathways (Figure 6). In our primary human hepatocyte model, CD40∶NADPH oxidase∶ROS initiated apoptosis and to a lesser extent necrotic cell death. The mode of hepatocyte cell death stimulated by hypoxia remains controversial. Both apoptosis and necrosis have been implicated in transplant models [30] consistent with our findings and in support of Lemasters proposal that a common mediator regulates both forms of cell death during hypoxia and H-R [31]. We propose that CD40∶CD154 activation in hepatocytes has at least three downstream consequences 1) initiation of apoptosis mediated via ROS 2) initiation of apoptosis mediated via FasL induction and 3) necrosis.

CD40 activation results in apoptotic death in many cells of epithelial lineage [7], [14]. Indeed, CD40 activation induces human hepatocyte apoptosis via up-regulation of FasL expression and subsequent Fas-mediated apoptosis [7]. Moreover, in human hepatocytes, it is the sustained activation of Activator Protein-1 (AP-1) and MAPKs, and not NF-kappaB, which is central to initiating apoptosis [32]. A similar signalling pathway has been reported in mediating cholangiocytes apoptosis after CD40 ligation [33]. Additionally, in cholangiocytes, CD40 mediated apoptosis also requires the activation of the JAK2-STAT3 pathway and the downstream activation of caspase-3 [14]. However, our study is the first to implicate ROS in this process of epithelial and specifically human hepatocyte, apoptosis. ROS can activate the MAPKs sub-families of JNK and p38, important regulators of cell death. Specifically, ROS can activate the MAP3K, apoptosis signaling kinase 1 (ASK1) resulting in phosphorylation of MEK 3/6 [34] and MEK 4/7 [34] which respectively activate p38 and JNK. Inhibition of p38 [35] and JNK [36] improves hepatocyte viability after hypoxia and during H-R in rodent models. We now show that CD40∶CD154 mediated human hepatocyte apoptosis is reduced by JNK inhibition and prevented by p38 inhibition. The precise mechanism of JNK mediated apoptosis is controversial. JNK can act upstream of mitochondria by activating the pro-apoptotic Bcl2 family [37] and also increases turnover of the anti-apoptotic protein c-FLIP [38]. p38 is thought to mediate apoptosis via phosphorylation of Bcl2 family members [39] and CD40-mediated-NADPH oxidase generated ROS is upstream of p38 activation in WEHI 231 cells [11]. Thus our data report for the first time a mechanistic link between CD40∶CD154 mediated ROS generation, MAPK activation and cell death. Taken together, these studies highlight the complexities of the upstream CD40 signaling pathway and suggest that CD40 can couple to at least ROS and AP-1 signaling pathways to regulate apoptosis. Whether CD40 ligation in human hepatocytes can also active the JAK-STAT pathways, as seen in cholangiocytes, remains to be ascertained.

CD40 ligation by CD154 is known to increase FasL expression resulting in autocrine/paracrine Fas-mediated apoptosis in human hepatocytes [7]. However, in contrast to *in vivo* studies [40], hypoxia and H-R did not result in increased hepatocyte FasL expression in our study. We confirmed that CD154 induced cell surface expression of FasL in human hepatocytes and showed for the first time that this is in part dependent on NADPH oxidase particularly during H-R. This suggests that other mechanisms are involved in regulating ROS-independent FasL expression. As discussed above, although CD40∶CD154 did not increase ROS during hypoxia, it did increase FasL expression although without a concomitant increase in apoptosis. However, the inhibition of mitochondrial ROS significantly reduced hepatocyte apoptosis during hypoxia. Fas mediated apoptosis in hepatocytes requires the downstream accumulation of mitochondrial ROS [41] and together with likely increases in hepatocytes antioxidant levels during hypoxia explains why we saw increased FasL expression in the absence of apoptosis in response to CD40 activation [4], [5]. This also suggests that different apoptotic signaling pathways may be initiated in response to CD40 activation under different conditions in distinct microenvironments.

Our data suggest that hypoxia, which precedes reoxygenation during preservation of an organ allograft for transplantation, can increase FasL expression thereby initiating liver damage before ROS generation during reperfusion has occurred. FasL is also increased on hepatocytes in chronically inflamed livers a situation characterized by tissue hypoxia. Thus the mechanism we describe is likely to operate in the chronic inflammation where large numbers of CD154 expressing effector cells can activate CD40 in a hypoxic environment [42].

We have previously reported that ROS generation can regulate human hepatocyte necrosis during hypoxia and H-R [2] and hepatocyte necrosis has been induced experimentally during hypoxia followed by reoxygenation [31]. We show that ROS generated by CD40∶CD154 not only induces hepatocyte apoptosis but also necrosis during normoxia and H-R. This process is NADPH Oxidase dependent as shown by the decrease in necrosis in the presence of DPI but in contrast to CD154-mediated hepatocyte apoptosis, necrosis is MAPK-independent. This suggests that NADPH oxidase generated ROS regulates divergent signaling pathways leading to both forms of cell death.

Therefore, coupled with previous work regarding CD40 mediated death of liver epithelial cells it is clear that the signaling pathways regulating this process are diverse. Clearly, in the present manuscript we demonstrate that CD154∶CD40∶ROS is an important activator of JNK and p38 which subsequently leads to cell death. However the JAK2-STAT3 is also important CD40 mediated apoptotic pathway [14] that may also be activated by ROS. Furthermore FasL is also up-regulated following CD40 activation. Therefore CD40 regulates multiple death signaling pathways in liver epithelial cells.

We have shown that macrophages and T-lymphocytes are an abundant source of CD154 in the chronically inflamed liver [43]. However platelets are also a rich source of CD154 [22] and within seconds of activation CD154 is translocated to the platelet surface then cleaved to produce soluble CD154. Platelets have been implicated in preservation injury during liver transplantation and in other conditions including immune mediated damage in hepatitis although the mechanisms of the effect are poorly understood [44], [45], [46]. We now show that activated platelets secrete functional CD154 that is capable of inducing ROS accumulation in human hepatocytes during H-R leading to hepatocyte apoptosis. This finding provides a novel mechanism to explain the effector function of platelets in liver injury and suggests that anti-platelet therapy together with anti-oxidants may be warranted not only to prevent reperfusion injury but also to reduce liver injury in chronic hepatitis and cirrhosis in which platelets are found in close proximity to hepatocytes [47], [48]. Although our findings suggest that platelets are a major source of CD154 capable of inducing injury as previously suggested [25] CD154 depletion from platelet-conditioned medium reduced but did not abolish human hepatocyte apoptosis suggesting either that there was residual CD154 activity in the PCM or that platelets release other active mediators of apoptosis [44].

In conclusion, we have shown that CD40∶CD154 mediated generation of ROS contributes to hepatocyte cell death by both apoptosis and necrosis in the hypoxic liver microenvironment. The ability of platelets as well as infiltrating T-lymphocytes and macrophages to deliver functional CD154 to hepatocytes suggests several potential mechanisms through which hepatocyte CD40 can be activated in the inflamed and hypoxic liver. The work supports therapeutic intervention with anti-platelet and anti-oxidant therapy in liver disease.
